# Supplementary material for: Patient-Derived Tumor Organoids to Model Cancer Cell Plasticity and Overcome Therapeutic Resistance
Source: Cells. 2025 Sep 18;14(18):1464. doi: 10.3390/cells14181464 (PMC12469014; doi:10.3390/cells14181464)
Supplement: Supplementary file 1 [file cells-14-01464-s001.zip › cells-3805060-supplementary-update.pdf]

Table S1. Experimental organoid-based models to study cellular plasticity.

Approach / Model	Source material / context	References
Patient-derived organoids (PDOs)	Tumor biopsies or resections (EOCRC, CRC, PDAC, breast, gastric, SCNEC, pediatric kidney) and normal tissues	[24–29,31,33–35,69–72]
Genetically engineered organoids	Murine intestinal epithelium engineered with APC, KRAS, TP53, SMAD4 mutations	[23,32,36]
	Murine intestinal epithelium engineered with APC, KRAS, TP53, TGFBR2 and/or SMAD4 mutations	[26,37]
	PDAC organoids engineered with KRAS, TP53, SMAD4, and CDKN2A mutations	[29]
PDX-derived organoids	Organoids established from tumors expanded in mice	[30]
In vivo implantation / xenograft of PDOs	PDOs implanted orthotopically, subcutaneously, or intraductally in mice	[23–27,30,33,36,37]
Co-culture systems	PDOs with CAFs, immune cells, or stromal niche factors	[29,31,32,35,70,72]
Modified culture assays (e.g., spheroid formation, SSFG assay)	PDOs dissociated into single cells or small clusters	[27,69]

EOCRC, early-onset colorectal cancer; CRC, colorectal cancer; PDAC, pancreatic ductal adenocarcinoma; SCNEC, small-cell neuroendocrine carcinoma; PDO, patient-derived organoid; PDX, patient-derived xenograft; CAF, cancer-associated fibroblast; SSFG, single-cell–derived spheroid formation and growth.
